# “On My Own, but Not Alone” - Adolescents’ Experiences of Internet-Delivered Cognitive Behavior Therapy for Obsessive-Compulsive Disorder

**DOI:** 10.1371/journal.pone.0164311

**Published:** 2016-10-06

**Authors:** Fabian Lenhard, Sarah Vigerland, Hedvig Engberg, Anna Hallberg, Hanna Thermaenius, Eva Serlachius

**Affiliations:** 1 Centre for Psychiatry Research, Department of Clinical Neuroscience, Karolinska Institutet, Stockholm, Sweden; 2 Stockholm Health Care Services, Stockholm County Council, Stockholm, Sweden; 3 Östersund Child and Adolescent Mental Health Service, Östersunds sjukhus, 831 83 Östersund, Sweden; Hospital Universitari de Bellvitge, SPAIN

## Abstract

**Introduction:**

Childhood Obsessive-Compulsive Disorder (OCD) is a prevalent and impairing condition that can be effectively treated with Cognitive Behavior Therapy (CBT). However, a majority of children and adolescents do not have access to CBT. Internet-delivered CBT (ICBT) has been suggested as a way to increase availability to effective psychological treatments. Yet, the research on ICBT in children and adolescents has been lagging behind significantly both when it comes to quantitative as well as qualitative studies. The aim of the current study was to describe the experience of ICBT in adolescents with OCD.

**Method:**

Eight adolescents with OCD that had received ICBT were interviewed with qualitative methodology regarding their experiences of the intervention. Data was summarized into thematic categories.

**Results:**

Two overarching themes were identified, autonomy and support, each consisting of three primary themes (self-efficacy, flexibility, secure self-disclosure and clinician support, parental support, identification/normalization, respectively).

**Conclusions:**

The experiential hierarchical model that was identified in this study is, in part, transferrable to previous research. In addition, it highlights the need of further study of important process variables of ICBT in young patient populations.

## Introduction

Obsessive-Compulsive Disorder (OCD) is characterized by recurring intrusive thoughts and ritualistic behavior [1] and affects about 2% of children and adolescents [2,3]. OCD is associated with severe impairments in academic, social and family functioning in the young person’s life [4]. As OCD in childhood is associated with an increased risk of future OCD as well as other psychiatric conditions [5–7], early detection and treatment is crucial.

The first hand treatment alternative for OCD is cognitive behavior therapy (CBT) [8,9]. CBT has been shown to be effective for about 70% of patients [10]. However, studies indicate that a majority of OCD sufferers do not get access to evidence-based treatments [11,12]. Consequently, innovative ways of disseminating effective treatments are needed to close the treatment gap.

Internet-delivered interventions have the potential to make evidence-based psychological treatments more accessible to patients, as those interventions are less bound to temporal and geographical barriers [13]. There is a growing body of evidence for internet-delivered cognitive behavior therapy (ICBT) with several reviews showing that ICBT is an effective treatment for adults with various psychological diagnoses [14–16]. The review of Hedman et al. found over 100 randomized controlled studies, demonstrating the substantial amount of evidence for ICBT in adult patient populations.

However, despite the fact that children and adolescents define the most active age group on the internet with daily access rates of up to 97% [17], research on ICBT for pediatric patient populations has been lagging behind significantly. To date, a comparatively small number of clinical ICBT trials have been published in the pediatric field, and although the findings indicate that ICBT appears to be promising in the treatment of children and adolescents with mental health problems [18,19], the evidence is limited at this point in time. For example, a review of ICBT for mood and anxiety disorders that included all ages found 52 studies of which only four included children and adolescents [15].

In the OCD field, a number of open trials and randomized controlled studies have demonstrated feasibility and efficacy of ICBT in adult populations [20–24]. However, in the childhood OCD field only limited evidence is available at this point in time. Our group published the first clinical trial of ICBT for pediatric OCD with 21 adolescents with clinical significant symptom severity [25]. All patients received a novel 12-week, clinician-guided ICBT intervention. Clinician-rated OCD symptoms changed significantly from pre to post-treatment and within-group effect sizes were in the large range with 48% of patients in remission at the post-treatment assessment. Patients rated the intervention as acceptable and most patients reported that they felt comfortable working with the internet format. The current qualitative study was conducted on a subsample of that earlier quantitative trial.

As little is known about how ICBT is experienced by younger patient populations it is important to gain patient perspective insights that complement quantitative results, especially in the child and adolescent ICBT field. One could hypothesize that the knowledge that is generated in the adult field only to a certain degree is transferable to younger patient populations due to important developmental differences. Qualitative methods could help us understand how interventions should be tailored with regard to cognitive, motivational, emotional and diagnosis-specific needs in different age groups. Currently, ICBT interventions for children and adolescents vary greatly in important characteristics, such as length of intervention, intensity, amount of therapist contact, amount of parental involvement, graphical design and technical solutions [19]. For example, intervention formats range from traditional approaches that translate face-to-face CBT into a web-based format [26] to innovative computer game-like solutions [27]. The design and technical functionality, as well as the content of the interventions, are likely to affect treatment process and outcome, however little is known about how those aspects are experienced by the children and adolescents that receive the ICBT treatments.

To summarize, while rigorously controlled quantitative trials are used as the gold standard design in the study of treatment efficacy, those studies often leave the ICBT intervention as a “black box” and generate little knowledge about the patient’s experience with the intervention. As ICBT for pediatric mental health problems is still to be considered in a developmental phase, more qualitative insights are needed to better understand the process that make patients accept, adhere to and engage with the interventions, in order to be able to design better treatments in the future.

The specific aim of this study was to extend earlier quantitative results and to analyze the experience undergoing ICBT for OCD from the perspective of adolescent patients with regard to meaningful experiential dimensions and potential areas for improvement of future ICBT interventions.

## Method

This study was part of a quantitative open trial study [25] that was registered at clinicaltrials.gov (NCT01809990). The study was approved by the Regional Ethical Review Board in Stockholm, Sweden. The protocol for this trial is available as supporting information; see S1 File. Further, this study is presented in accordance with COREQ standards (S2 File), which is a reporting standard for qualitative research, taking into account three domains of study presentation: research team and participants, study design with focus on theoretical framework, participant selection, setting and data collection, and analysis and findings [28].

### Participants

**Fig 1:**
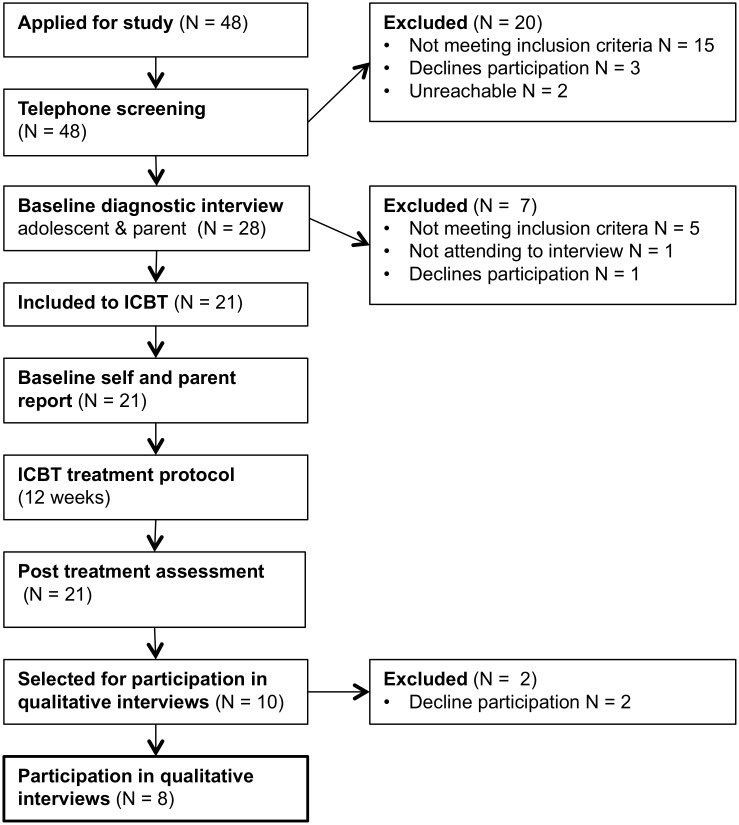
Study flow chart.

The participants in this study were a sub-sample of participants in an open trial on ICBT for adolescents with OCD [25], see Fig 1. Participants were recruited during January to February 2013 through advertisements in local newspapers, schools and mental health care units in the Stockholm area. Via an online registration form, parents or adolescents could register their interest in the study. Applicants were then called by a psychologist for a broad screening of inclusion and exclusion criteria via telephone. In a next step, the adolescents were invited to a face-to-face assessment together with their parents for a more thorough assessment of study eligibility. Inclusion criteria were: a) a primary DSM-IV-TR [29] diagnosis of OCD, b) a total score of ≥ 16 on the Children’s Yale-Brown Obsessive-Compulsive Scale (CY-BOCS [30]), c) age between 12 and 17 years, d) ability to read and write Swedish, e) daily access to the Internet, f) a parent that was able to co-participate in the treatment, and g) for participants on psychotropic medication: a stable dose for the last 6 weeks prior to baseline assessment. Exclusion criteria were: h) diagnosed autism spectrum disorder, psychosis, bipolar disorder, severe eating disorder, i) suicidal ideation, j) on-going substance dependence, k) not able to read or understand the basics of the ICBT self-help material, l) to have completed a course of CBT for OCD within last 12 months, m) on-going psychological treatment for OCD or another anxiety disorder. Patients fulfilling all inclusion criteria and no exclusion criteria were offered participation in the study and provided with an informed consent study participation form that was signed by the adolescent and parent or primary caregiver.

Twenty-one adolescents were included and received the ICBT intervention. Of those, 18 initially agreed to be contacted for participation in the qualitative interviews. A sample size of six to ten participants was deemed an appropriate sample size for the planned qualitative analyses and a random set of ten adolescents were contacted during the follow up period after ICBT (3 to 6 months after treatment) and asked if they were willing to participate in the interviews. Of those, eight adolescents accepted and two declined participation. A separate consent form for participation in the qualitative study was provided and signed by adolescents and parents. Eight interviews were conducted in October to November 2013. As no new themes emerged the data collection was considered completed at this point. Study participants were also found to represent a credible combination of characteristics regarding age, sex, pre-treatment OCD symptom severity and comorbidity, degree of treatment completion and responder status. See Table 1 for characteristics of the eight study participants.

**Table 1:** Study 1 participant characteristics.

Participant	Sex	Age	Pre-treatment CY-BOCS score	Comorbid diagnoses	Completed treatment chapters	Treatment responder
1	female	12	18	Social phobia, specific phobia, GAD	6	no
2	female	13	18	Separation anxiety disorder, GAD	7	no
3	female	14	23	-	12	yes
4	female	15	24	Specific phobia, GAD	12	yes
5	male	12	24	Depression, specific phobia	10	no
6	male	13	21	-	4	yes
7	male	16	18	-	9	yes
8	male	17	22	Specific phobia	12	no

*Note*: CY-BOCS = Children’s Yale-Brown Obsessive Compulsive Scale (a score of 16 or above indicates moderately severe OCD symptoms), GAD = Generalized Anxiety Disorder.

### Intervention

The OCD treatment intervention “BiP OCD” is delivered via a secure technical Internet platform that enables the age-appropriate presentation of different treatment content to pediatric patient populations. The platform has been used in several clinical ICBT trials for children with anxiety disorders [31,32], irritable bowel syndrome [33] and OCD [25]. Patients log in via personal user names and passwords to access the treatment content. The ICBT treatment content is presented in chapters, much like chapters in a self-help book. The chapters contain psychoeducative texts, films and CBT exercises, such as exposure exercises and cognitive interventions. Patients have regular contact with an experienced clinician (usually a trained psychologist) via messages that can be sent inside the treatment platform (resembling email). The clinician can also directly comment on exercises that the patient has been working on, and can therefore give specific feedback, support and motivate the patient. In this way a patient usually has contact with his or her treating clinician several times a week. Parents are involved in the treatment via a separate parent login with parent-specific content, such as coping strategies and tips on how to support the child’s treatment in a constructive way.

Regarding treatment content, BiP OCD consists of evidence-based CBT components that are usually represented in OCD treatment manuals, namely psychoeducation about OCD and its self-sustaining nature, exposure and response prevention, cognitive techniques and relapse prevention. A more in-depth description of BiP OCD has been published elsewhere [25].

### Data collection

The data collection in this study was conducted according to the interview method defined by Seidman et al. [34]. Participants were interviewed at post-treatment during the period October–November 2013. The interviews were conducted by two trained psychologists (HT and AH) and took about 30–60 minutes per participant. All interviews were taped and transcribed. Due to the amount of verbal material generated (in total 4 hours 48 min of taped material, or 16.036 words of transcribed text), experienced interviewers, good sample specificity in relation to the research question, and a thematic in-depth analytic strategy, a sample size of six to ten participants was deemed appropriate and in line with recommendations for phenomenological qualitative research [35,36]. Five of the interviews were conducted at our clinical research unit, two at the participants’ homes and one on the telephone. Participants were allowed to choose the place of interview in order to facilitate participation.

The interview approach and questions were developed according to recommendations on phenomenological qualitative methodology [34]. The questions were aimed to cover four general areas of treatment experience (S3 File):

1. general positive and negative experiences
2. experiences of the treatment process
3. experiences of the usefulness of the treatment
4. unmet expectations and suggestions for improvement

One initial open question per area was posed to the participant and then followed up by subsequent, in-depth interviewing in order to yield good understanding of the individual participants’ experiences with the program. All participants were informed about that the main aim of the study was to describe their individual experiences with the Internet treatment. Furthermore, participants were informed that their information would be presented in an anonymous format, not allowing identification of single individuals.

### Data analysis

The data in this study was processed according to the thematic analysis method described by Schilling [37] which is a systematic approach of analyzing verbal and textual data. Thematic analysis has previously been used in the study of internet-delivered interventions e.g. smartphone-based treatment for depression [38] and internet-delivered CBT for procrastination [39].

All interviews were taped and transcribed for further analysis. The interviews were analyzed by two senior psychologists experienced in thematic analysis (HT and AH). Raters had not been exposed to the BiP OCD intervention or its development prior to the study. In a first step raters read and analyzed the transcripts separately in order to condense and structure the data according to the conceptual framework of the study (i.e. finding experiential categories). In a second step the raters jointly structured the material to compare individually identified themes, resolve any redundancies and modify themes in order to achieve best possible credibility and transferability. As part of this process, initially identified themes were iteratively tested and compared against the raw material of the transcribed interviews. To further refine the results, each general theme was hierarchically differentiated into sub-themes. After processing eight interviews in this manner no new themes emerged.

Finally, the identified themes were labeled with descriptive titles and exemplified with original quotes from the transcripts to give a more in-depth understanding of how the experiential themes relate to the raw data.

## Results

**Fig 2:**
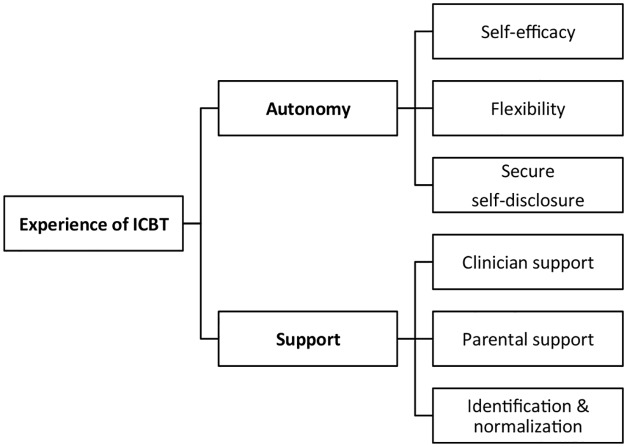
A hierarchical model of adolescents´ experiences of ICBT.

The resulting hierarchical model represents the patients’ experiences of ICBT on two overarching themes, autonomy and support, each consisting of three primary themes: self-efficacy, flexibility, secure self-disclosure and clinician support, parental support, identification/normalization, respectively (Fig 2).

### Autonomy

The overarching theme of autonomy describes the experience of a working format that allowed a relatively independent, demand-driven process and a high degree of responsibility for the treatment on the patient’s side. Patients felt that they were in control of how and when to do the treatment, at which pace and intensity, and that the Internet format was a secure way of expressing thoughts and feelings. The overarching theme of autonomy consists of the three primary themes self-efficacy, flexibility and secure self-disclosure.

#### Self-efficacy

The term self-efficacy is understood as the individual’s belief in the own ability to cope effectively with situations and problems. The participants described the positive experience of working independently with the treatment material and by that having some degree of control over the therapy process. Through the strategies that were given in the treatment the patients not only experienced to learn effective coping behaviors but also that they themselves had the responsibility to carry out the treatment, with support of the parents and the clinician. This experience was seen as a contrast to being fully dependent on a parent or clinician in face-to-face treatment. Generally, this relative independence from adults was seen as a positive aspect and as one that strengthened the results and long-time benefits of the treatment, promoting a sense of self-efficacy.

“If you work with a parent you can get support, but at the same time it can be good to work a bit on your own as well, so you can stand on your own feet a little.”

#### Flexibility

Several participants experienced advantages of being able to choose when and where to work with ICBT. The treatment was experienced to be available when needed. In comparison, it was seen as both time and energy consuming to commute to a local clinical unit, and at the same time miss school or leisure time activities. Therefore, Internet treatment was generally preferred in order to being able to prioritize other activities in life, as well as fitting in the treatment into the weekly schedule.

”It was good to be able to do it at home, it would have been tough if I would have needed to miss school time. Here I could decide by myself, now I want to practice, and I did not have to wait.”

As well, the possibility of being able to communicate questions or problems when they arose was seen as positive. Having been able to choose when to work with the treatment was experienced as positive for motivation and facilitated focus on important tasks.

“If you would have to go to a meeting every week you would need to… write it down in order to not forget until next time you go there. I think this was very good. Going to a psychologist every week, that’s not practical. I don´t think it would work better.”

#### Secure self-disclosure

The theme of secure self-disclosure was experienced in relation to the internet clinician and describes the patient’s willingness to communicate information about him- or herself, such as thoughts, feelings, doubts, failures, successes and fears, and at the same time feeling secure about doing so. Working with the ICBT intervention was experienced as a possibility to communicate about OCD and related problems in everyday life. The participants expressed that the Internet format facilitated self-disclosure, compared to how they imagined a face-to-face contact would have been like.

“I think you dare more when sitting at the computer, when you don´t have to go and visit somebody. It is good that it is on the Internet, you can do it alone and be honest.”

### Support

While the participants highlighted the advantages of an autonomic working format, a complimentary theme was found in which the importance of support from others was expressed. The aspect of support was identified as an overarching theme that summarizes the three primary themes: clinician support, parental support and identification / normalization.

#### Clinician support

The clinician support appeared to have an important role in the adolescents’ experiences of the treatment. The participants were aware that their work and progress was monitored by a clinician and that the clinician reviewed and commented on their working exercises.

”He [the clinician] said that it went well, that he saw that I worked with it. So that was positive, that I was not doing this on my own. Many of my compulsions are not noticeable for others, so if I stopped doing them it was not a big difference for others. So, that was nice that he [the clinician] knew that I had made progress.”

The availability of the clinician via messages in the Internet treatment platform was appreciated and experienced as a positive way of support. Also, being able to send a message to the clinician whenever problems or questions arose gave an impression of being in permanent contact with the clinician.

“It was like, ok now I wonder about this, I’ll write [a message] right away. It felt like he was logged in like 24–7. He is there and has the computer on all the time in case you write something.”

Regarding content, the clinician messages and comments were experienced as appropriate help when requested. There was also a motivational aspect involved in the clinician support that helped the participants to continue working with the treatment.

”I received the support I needed. I felt that she [the clinician] was on my side and that I was able to do it [the treatment].”

As part of the study routines participants met their treating clinician during baseline assessments. It was experienced as important to have met the clinician personally before starting the Internet treatment, knowing who the clinician was and having made a first impression.

“That way you could really be sure that it was a real person, so you know a little who it is. When you write you know that it´s not a robot that answers. That was an advantage in my opinion.”

While the internet-based clinician contact was generally experienced as preferable over a full course of face-to-face treatment, a sub-group of participants described a preference of additional face-to-face contact in order to follow-up, clarify certain aspects and give additional individually-tailored support.

“We [mother and participant] talked about that we could have met the psychologist and talked about how things were going a little more often. Maybe three to four times during that period. I would have liked to talk about how things were going, if there was something I did not understand, if it got better or worse, for example.”

#### Parental support

The participants highlighted the advantages of parental involvement in the intervention. The participants experienced that they got motivated by their parents as well as it was appreciated that parents reminded and supported them in working regularly with the treatment. No participant experienced the involvement of parents as a negative aspect.

”It was good to do it together with parents, that they were involved so I did not feel alone. They were involved, but I did most of it on my own.”

Another aspect that was described by the participants was better communication about their symptoms as a result of parental involvement in the treatment, as parents and adolescents received similar information about OCD and CBT strategies.

”Earlier, I didn’t like to talk about it, that I had obsessions. Thought it was embarrassing. It is not fun to have obsessions. But then, when we started with the project it was more like… it was not like it was as embarrassing anymore. If I had done it by myself I would not really have talked with my mom about it.”

#### Identification and normalization

By participating in the treatment and reading e.g. case examples and psychoeducative texts patients understood that there are others with the same symptoms and problems. Identification with the case examples was found helpful in understanding the nature of OCD. Furthermore, by reading the treatment material the participants describe that their symptoms are experienced as more normal and less divergent.

“They gave examples about different kinds of OCD. You understand that you are not completely insane. There are other people who… But also how they had worked with it. That was interesting to read about.”

## Discussion

The aim of this study was to describe adolescents’ experiences of an ICBT intervention for OCD. The thematic analysis of participant interviews three to six months after the treatment resulted in a hierarchical model with two overarching themes and six primary themes. Conceptually, the overarching themes can be understood as complimentary aspects, with autonomy expressing the patient’s positive independence with regard to responsibilities for the ICBT treatment, and the theme of support representing practical and psychological resources that enable the adolescent patient to age-appropriately express this autonomy. In other words, one could assume that ICBT without support could possibly be experienced as solitude, and in turn, support without autonomy might possibly be experienced as over-involvement or restriction of freedom. The results therefore suggest that both support and autonomy are needed for a positive experience of ICBT.

Generally, our overarching themes correspond to results from a meta-synthesis by Knowles et al. on qualitative dimensions of computerized therapy for depression and anxiety [40]. In this study eight qualitative studies of computerized therapy were reviewed and summarized. Two overarching dimensions were identified, of which one dimension had the endpoints “expressed support” and “empowerment”, conceptually similar to the overarching themes of the current study.

More specifically, the concept of autonomy had previously been identified as an important experiential dimension of internet-delivered therapy. For example, an earlier qualitative study on ICBT for depressed adult patients identified autonomy as one of the core experiential dimensions of ICBT [41]. As in our study, the flexibility of working with ICBT at a self-chosen time and place was found an important feature in creating the experience of autonomy. Likewise, temporal and practical flexibility has been found an important experiential dimension in ICBT treatment of adults with eating disorders [42]. However, flexibility to schedule ICBT themselves has also been described as a challenge for patients, as there is a risk of competing everyday activities, and self-discipline and motivational factors may become a treatment barrier for some patients [39,42]. Such challenges were not identified in our study, which in part could be due to the parental support that our adolescent patients had during the intervention, and thus highlighting the importance of social support for adherence to ICBT. To summarize, autonomy seems to be a central experiential theme of ICBT, both in adults and adolescents, and it appears that the flexible format of ICBT promotes autonomy in the patients.

Similarly, the theme of support has been highlighted in previous studies, as the earlier mentioned study of depressed adults showed, where one dimension called “belonging” was identified, summarizing support from friends, partners and family during the intervention, as well as one dimension “recognition and self-identification”, which summarized identification with the treatment material [41]. Those descriptions mirror the support theme that was found in our study. More specifically, the finding of clinician support as an important experiential aspect of ICBT is also supported by quantitative meta-analyses on ICBT for adults showing that interventions that involve clinician support are associated with larger effects compared to interventions without any clinician support, e.g. self-guided interventions [43,44]. Furthermore, clinician support has been found to increase patient adherence to ICBT in adults with anxiety and depression [45]. Again, little data is available for the child and adolescent ICBT field, which underlines the importance of additional efforts to further understand the importance of social support when treating this age group via the Internet.

One of the suggestions for improvement made by the participants was the combination of ICBT with face-to-face CBT. A”blended” format like this has been tested before, e.g. for adults with depression and anxiety [41,46], but to our knowledge, to date there is only one such study in the child ICBT field [47]. Such a blended format would of course increase direct social support, and one could hypothesize that some patients would appreciate more direct contact and feedback from clinicians, while maintaining a certain degree of the internet-delivered format. This could be a way to more straightforwardly target motivational issues, difficulties of scheduling ICBT at home and give appropriate support to those patients that might have difficulties expressing themselves in writing. Qualitative investigations of blended approaches are clearly warranted.

### Limitations

This study is limited by its qualitative design and transferability is therefore restricted to patients in the current context and age group. In order to yield conclusions that are transferrable more generally, the results should be replicated in other contexts, ages and patient groups.

Secondly, the study results are limited to the patients’ views of ICBT, and parent and/or clinician variables have been omitted. A more complex picture would probably evolve if the experiences of parents and clinicians had been investigated and more could have been learned about the interaction of all three perspectives. However, we believed that the patient experience was the most relevant variable, and involvement of more perspectives would have made data analysis difficult to manage.

Thirdly, qualitative research is sensitive to interviewer bias. The two interviewers in this study were not previously involved in the construction of the treatment or data collection process, nor were they clinicians in the clinical trial. However, both were experienced clinical psychologists and preconceptions about ICBT, CBT or psychotherapy in general could have influenced the data analysis. Though raters were experienced in qualitative research, this is a source of bias that often is built-in in qualitative methodology as such. Therefore the results of this study have to be validated against other studies, both qualitative and quantitative, in order to demonstrate sufficient dependability.

### Future directions

The themes that were identified in this study are in part congruent with previous research. However, as we have described, most of the knowledge on the role of experiential themes of ICBT is generated in the adult literature. It is therefore necessary to generate more qualitative knowledge in the child and adolescent ICBT field, in order to gain better insights of how patients react to and engage with those relatively new interventions, and which further improvements can be made. A methodically sound way to further validate the results of the current study would be to apply quantifiable operationalizations of the primary and overarching themes and to empirically test whether those themes mediate or predict treatment adherence, engagement and outcome in clinical trials of ICBT for children and adolescents. In this way we could generate more knowledge of the hypotheses that were generated in this study, as well as statistically testable results of the model that has been presented. Moreover, key variables could be experimentally manipulated, e.g. amount of clinician or parent support, in order to test their specific contribution to ICBT.

## Conclusion

In this qualitative study of adolescent patient experiences of ICBT for OCD, we found a hierarchical model with the overarching experiential themes autonomy and support. The results are, in part, confirmed by previous research. As most of the available evidence has been generated in the adult ICBT field, further qualitative and quantitative research is needed to verify the model that was presented in this study.

## Supporting Information

S1 File Study Protocol. (PDF)

S2 File COREQ Checklist. (PDF)

S3 File Interview questionnaire. (DOCX)
