# Supplementary material for: “On My Own, but Not Alone” - Adolescents’ Experiences of Internet-Delivered Cognitive Behavior Therapy for Obsessive-Compulsive Disorder
Source: PLoS One. 2016 Oct 6;11(10):e0164311. doi: 10.1371/journal.pone.0164311 (PMC5053512; doi:10.1371/journal.pone.0164311)
Supplement: S3 File — (DOCX) [file pone.0164311.s003.docx]

**Intervjuguide BiP OCD kvalitativ studie**

| **Område** | **Startfrågor** |
| --- | --- |
| Positiva och negativa upplevelser | Vad tyckte du om / inte om i programmet? |
| Upplevelser av behandlingsprocessen | Hur gick det till när du arbetade? |
| Upplevelser av behandlingens användbarhet | Vad i programmet var användbart/hjälpsamt för dig? |
| Otillfredställda förväntningar och förslag för förbättringar | Hur skulle ”drömhjälpen” se ut? |

**Interview question areas BiP OCD qualitative study**

| **General area** | **Start questions** |
| --- | --- |
| general positive and negative experiences | What did you like / not like about the program? |
| experiences of the treatment process | Could you explain how you worked with the program? |
| experiences of the usefulness of the treatment | What parts of the program were useful / helpful for you? |
| unmet expectations and suggestions for improvement | Was there anything else that you would have wished for? |
